# Mr. Hardy, on Failure of Cow Pock Inoculation

**Published:** 1808-09

**Authors:** Thomas Hardy

**Affiliations:** Walworth


### To Dr. BRADLEY.

DEAR SIR,

HAVING practiced vaccination during the last eight years, with results invariably satisfactory until this time, it is with feelings of somewhat a painful nature, that I submit to your notice the following detail of a case, in which the Cow Pock failed to preserve my patient from receiving the infection of Small Pox in the natural way.

As I have no cause to support but that of truth, I see no reason why this occurrence should not be recorded in the Medical and Physical Journal, although it will add one more proof to the position, that cow-pock will not *universally* protect the constitution from the contagion of the small-pox. And yet, I believe the protection afforded by vaccination to be so *general*, as to rank the discovery amongst the most important and useful which are to be found in the history of medicine.

I am, &c.

THOMAS HARDY.

*Walworth, Aug.* 13, 1808.

---

April 2, 1804, I vaccinated Catharine Dawson, aged 10 weeks, with matter received from the Central House of the Royal Jennerian Society. The virus inserted in the left arm did not produce any effect; but that applied to the right arm was followed by the usual appearances. On the sixth sixth and seventh days after inoculation, the inflammation surrounding the vesicle was extensive, and there was rather more constitutional febrile affection than I have generally observed in cow-pock.

June 24, 1808, I inoculated the infant brother of Catharine Dawson with small-pox, and the child had a plentiful eruption of distinct pustules. Catharine, as well as another child of Mr. Dawson, nearly two years old, and vaccinated by me May 1, 1807, had free access to their brother all the time that he laboured under small-pox. On Sunday, July 17, Catharine was much indisposed, her skin hot and dry, eyes heavy, and bowels constipated. She continued ill until the Thursday following, when a considerable eruption appeared about the breast and stomach, and which I supposed to be chicken-pock, until the fourth day of the eruption, when the phenomena, both local and general, induced me reluctantly to alter my opinion. On the fifth, sixth, seventh, and eighth days of the eruption, the patient was seen by several professional friends; amongst others, by Doctors Adams, Walshman, Walker, and Lidderdale, all of whom pronounced the disease to be small-pox. And after examining the child's arm, declared the cicatrix to exhibit the exact appearance produced by vaccination.

Nothing peculiar occurred in the further progress of the disease. I inoculated the child, vaccinated last year, with matter taken from Catharine, but she has resisted not only the infection produced in her brother's case, but that of the small-pox virus inserted in both her arms. One of the punctures not inflaming at all, and the other exhibited only a slight degree of inflammation until the eighth day, which then suddenly disappeared, having been unaccompanied with pyrexia, or other symptoms of indisposition.

